# A longitudinal study on the impact of subjective exclusion on changes in self-esteem: the mediating effect of perceived income inequality

**DOI:** 10.1371/journal.pone.0321271

**Published:** 2025-04-02

**Authors:** Wan-Kyeong Park, Soo-Bi Lee

**Affiliations:** 1 Department of Social Welfare, Korea Nazarene University, Cheonan, Chungcheongnam-do, Republic of Korea; 2 Department of Social Welfare, Daejin University, Pocheon, Gyeonggi-do, Republic of Korea; Middle Tennessee State University Jennings A Jones College of Business, UNITED STATES OF AMERICA

## Abstract

This study explored the impact of the subjective exclusion perceived by adults on the development trajectory of self-esteem and verified the mediating role of perceived income inequality. We analyzed data from the 2017–2020 Korea Welfare Panel Study on 1,410 20–59-year-old research participants. Descriptive statistics were used to analyze the key variables and research participants’ general characteristics. A structural equation model investigated how the change trajectory of self-esteem and subjective exclusion affected the development trajectory of self-esteem via perceived income inequality. First, greater perceived subjective exclusion is associated with higher perceived income inequality, which acts as the mediator variable. Furthermore, the higher the perceived subjective exclusion, the lower the initial self-esteem; this gap increased over time. Second, perceived income inequality did not significantly impact initial self-esteem but positively significantly affected self-esteem’s rate of change, showing a wide variation. These findings suggest the necessity of social support systems and policy interventions to restore self-esteem, a cornerstone of mental health, among individuals experiencing social exclusion. Additionally, given that perceived income inequality impacts self-esteem, efforts to address social structural inequalities should be strengthened alongside clinical interventions.

## 1. Introduction

Social exclusion is defined as the process in which individuals or groups are completely or partially excluded from the society to which they belong [1]. It encompasses multiple deprivations such as low income, unstable job situations, poor quality housing, family stress, and poverty [2]. Social exclusion provides a more nuanced interpretation of poverty issues and has been extensively discussed in recent years [3]. It is also crucial in understanding physical health and psychological well-being [4,5].

Social exclusion encompasses negative experiences, marginalization, and deprivation, which can impact various aspects of an individual’s life over time. Therefore, a simplistic approach to understanding an individual’s experience of exclusion based solely on objective indicators may hinder a closer examination of the level and context of exclusion. Park et al. [3] highlighted the importance of taking a holistic approach to understanding an individual’s current life situation and experiences through their subjective perceptions and feelings about the exclusion they are facing. This perspective underscores the significance of such experiences and perceptions in assessing exclusion, as argued by Veenhoven [6], who proposed a distinction between objective and subjective exclusion.

Veenhoven [6] explained that objective exclusion is characterized by observable indicators and explicit criteria, whereas subjective exclusion is self-reported based on implicit criteria. The measurement of subjective exclusion relies on self-reporting, wherein individuals evaluate their level of exclusion based on their own perceptions and knowledge [7]. This self-reported method serves as a valuable tool for measuring subjective exclusion. For example, a low bank account balance may be considered objective exclusion, while an individual’s perception of their wealth as insufficient can be deemed subjective exclusion [6].

Subjective exclusion refers to exclusion from various domains of social life, leading to the denial of rights or access to capital [8]. Consequently, this concept is particularly useful for examining aspects that are challenging to assess in objective terms. Social exclusion can negatively impact not only mental health, such as depression and social anxiety [9], but also self-esteem [10]. This process typically unfolds in stages: when individuals fail to recover from persistent experiences of social exclusion, the psychological resources that support resilience gradually diminish, ultimately leading to resignation [11]. In other words, chronic exposure to social exclusion can result in helplessness and depression, ultimately lowering self-esteem [11,12].

Meanwhile, experiences of exclusion appear to negatively impact income and poverty [13,14], potentially leading to a vicious cycle, as it can exacerbate income inequality and further deepen issues of class polarization. Osborne et al. [15] found that the perception of one’s status relative to others serves as a mechanism linking income and inequality. Tadjoeddin [16] highlighted that exclusion experiences are associated with low-income populations, rural areas, informal economic activities, women, and individuals with disabilities, leading to economic disparities between high- and low-income groups.

Social exclusion restricts individuals from participating in social, economic, cultural, and political life, ultimately weakening their social status and identity [17]. This exclusion is often accompanied by limited material income, restricted participation in social activities, and a sense of psychological detachment from mainstream society [18]. These characteristics are closely associated with socioeconomic problems such as unemployment, poverty, and income inequality [19,20]. In fact, previous studies have reported that social exclusion serves as a factor that exacerbates these problems [21,22].

Experiencing exclusion often leads to social isolation, which subsequently weakens social networks and depletes psychological resources. Kim [23] pointed out that individuals who experience social exclusion and become socially isolated tend to develop an increased fear of job-seeking. As a result, they may end up working in low-wage, insecure jobs with poor working conditions. These experiences of exclusion heighten perceptions of income inequality and, consequently, may negatively affect self-esteem trajectories.

This pattern is significant as it not only widens social status gaps but also intensifies perceptions of income inequality. These perceptions, in turn, contribute to serious psychosocial consequences, including depression, anxiety, and diminished social trust [24–27]. Given the profound relationship between exclusion, income inequality, and their potentially severe quality-of-life implications, more comprehensive research, public discourse, and policy interventions are necessary to address these issues.

Moreover, income inequality acts as a risk factor that diminishes an individual’s self-esteem. Previous research indicated a mutually negative relationship between these two variables [28–31]. Self-esteem, broadly defined as an individual’s overall positive self-evaluation [32], is crucial, as high self-esteem can positively impact individuals and the society [33]. While numerous studies explored the correlation between poverty and self-esteem, emphasizing the idea that high self-esteem can mitigate psychosocial challenges even in the face of poverty [34–37], limited research has delved into the link between social exclusion and self-esteem as an alternative to poverty.

Considering the potential of high self-esteem to enhance life satisfaction, quality of life, and coping mechanisms during crises [38], it is crucial to examine this concept in the context of negative experiences such as social exclusion and deprivation. Hence, this study sought to verify how perceived income inequality mediates the relationship between subjective exclusion and the development trajectory of self-esteem.

## 2. Methods

### 2.1. Research targets

This study utilized data from the 12^th^ to the 15^th^ Korea Welfare Panel Study (KOWEPS; 2017–2020). The KOWEPS, conducted by the Korean Institute of Health and Social Affairs in conjunction with the Social Welfare Research Institute of Seoul National University, is an ongoing National Approval Statistics (No. 33109) of nationally representative South Korean households. The KOWEPS is a longitudinal panel survey that ensures nationwide representativeness [39]. Accordingly, this study used secondary data. Specifically, this study focused on adults aged 20–59 years, encompassing both young and middle-aged individuals for individual analysis. After excluding missing values for key variables, responses from 1,410 participants were included in the analysis.

### 2.2. Measurement tools

#### 2.2.1. Subjective exclusion.

The latent variable of subjective exclusion was measured by assessing satisfaction levels in various areas of multidimensional life. This study employed the subjective exclusion index used in Park and Jang’s methodological study [7]. This index consists of satisfaction in the areas of residential environment, interpersonal relationships (including social and family connections), leisure activities, and subjective health. Each area of subjective exclusion was evaluated using a 5-point Likert scale (“1 = very good” to “5 = very unsatisfied”), with higher scores indicating higher levels of dissatisfaction, thereby suggesting a stronger sense of exclusion for the individual.

#### 2.2.2. Perceived income inequality.

In relation to perceived income inequality, this study utilized welfare perception-related questions from the supplementary survey of the KOWEPS. The specific question asked, “In your opinion, how equal is Korea in terms of income and wealth?” Respondents were asked to select a score ranging from one to seven points. A higher score indicates a higher level of perceived income inequality.

#### 2.2.3. Self-esteem.

Self-esteem was assessed using the 10 questions from Rosenberg’s self-esteem scale [40], rated on a 4-point Likert scale (“1 = usually not” to “4 = always”), with the total sum of points used for analysis. Among these questions, five items related to self-esteem (e.g., “I am a valuable person,” “I have a good personality,” “I can work well with others,” “I have a positive attitude,” and “I am generally satisfied”) were reverse coded. Conversely, scores for questions such as “I am a failure,” “I feel like I have nothing to be proud of,” “I wish I could respect myself,” “I feel like I am useless,” and “I think I am not a good person” were summed as they were originally measured.

The self-esteem variable utilized in the growth model was calculated by aggregating the scores from each of the four years of data collected from the welfare panel. Reliability of the self-esteem scale was found to be.808 in the first year,.811 in the second year,.826 in the third year, and.810 in the fourth year, with all Cronbach’s alpha values exceeding 0.8.

#### 2.2.4. Control variables.

The control variables in this research were categorized based on 60% of the median income, using the Organisation for Economic Co-operation and Development’s household equivalence scale of the relative poverty concept. These data were obtained from the KOWEPS. The input variables included poor households (1) and general households (0). Education level was treated as a continuous variable in the analysis.

### 2.3. Analysis method

This study used a structural equation model to confirm the trajectory of change in adults’ self-esteem and to examine how subjective exclusion impacts the development of self-esteem through perceived income inequality. Prior to conducting the structural equation model, frequency analysis and descriptive statistics were performed to determine the sociodemographic characteristics of the participants and the key features of the variables using SPSS 26.0. Subsequently, the structural equation model analysis was conducted using AMOS 26.0 to identify a suitable latent growth model (LGM) related to the development of self-esteem and to validate the research model. The maximum likelihood method, which assumes multivariate normality of the research model, was utilized, and model fit was assessed using χ^2^ as an indicator of overall fit, along with the Tucker-Lewis index (TLI) and comparative fit index (CFI) as relative fit indices and root mean square error of approximation (RMSEA), goodness-of-fit index, and RMR as absolute fit indices. Generally, an RMSEA index between.05 and.08 is considered appropriate, along with TLI, CFI, and normed fit index values higher than.90 [41]. In contrast with cross-sectional studies that focused on observations at a specific time point, the LGM allows for the identification of dynamic changes occurring at multiple time points based on longitudinal data that are measured repeatedly over more than three time points. Thus, the LGM is valuable for understanding longitudinal changes over time [42].

### 2.4. Ethical considerations

The raw data used in this study, the Korea Welfare Panel Study (KOWEPS), are classified as National Approval Statistics (No. 33109) and were collected after receiving approval from the Institutional Review Board of the Korea Institute for Health and Social Affairs. As a result, these data are publicly accessible. Therefore, ethical approval was not required for this study. Informed consent from participants was not obtained, as all data were anonymized before analysis. All procedures contributing to this research comply with the ethical standards of the relevant national and institutional committees on human experimentation and align with the Declaration of Helsinki.

## 3. Results

### 3.1. Sociodemographic features of the research targets and general characteristics of the main variables

The demographic characteristics of the 1,410 participants in this study are summarized as follows (see Table 1). In terms of gender, 43.0% were male and 57.0% were female. Regarding age distribution, 30.4% of the participants were in their 20s and 30s, categorized as the younger age group, while 69.6% were in their 40s and 50s, representing the middle-aged group. Educational attainment showed that 53.9% had a high school education or less, while 46.1% had completed a college education (including associate degrees). Geographically, 46.0% of the participants resided in the capital region, and 54.0% were from non-capital regions. Marital status was distributed as follows: 16.2% were unmarried, 73.3% were married, and 10.5% were either divorced, widowed, or categorized as others. Employment status indicated that 76.9% were employed, while 23.1% were unemployed. Lastly, in terms of income status, 11.2% belonged to low-income households, and 88.8% were from general-income households.

**Table 1:** Sociodemographic characteristics of research targets.

Variables	Division	Frequency	Percentage
Gender	Men	606	43.0
	Women	804	57.0
Age (years)	20s–30s	428	30.4
	40s–50s	982	69.6
Education level	High school graduate	760	53.9
	Community college graduate	234	16.6
	Beyond university graduate	416	29.5
Region	Capital region	648	46.0
	Non-capital region	762	54.0
Marriage status	Single	228	16.2
	Married	1,034	73.3
	Divorced/Bereaved/Others	148	10.5
Occupation status	Employed	1,084	76.9
	Unemployed	326	23.1
Low-income status	Applicable	158	11.2
	Non-applicable	1,252	88.8
Total		1,410	100.0

Regarding the main variables, for the four-year average of self-esteem, the results showed a slight annual increase in mean scores: Year 1 (M =  3.15), Year 2 (M =  3.16), Year 3 (M =  3.19), and Year 4 (M =  3.18). Further, this study analyzed the descriptive statistics of the independent variables in the research model. First, in relation to subjective exclusion, the means of subjective economic satisfaction, family income satisfaction, subjective health status, social relationship satisfaction, family relationship satisfaction, and leisure life satisfaction were assessed on a five-point satisfaction scale in the welfare panel. The results showed that subjective economic satisfaction (M =  2.98) received the highest score, while family relationship satisfaction (M =  2.01) received the lowest score. The perceived income inequality was relatively high, with an average of 3.84 points. All variables exhibited skewness values of 3 or lower in absolute value and kurtosis values of 10 or lower in absolute value, indicating that the assumption of normality was met (see Table 2).

**Table 2:** General features of main variables.

Variable		Mean	Standard deviation	Skewness	Kurtosis
Self-esteem	Year 1	3.15	.35	-.642	1.35
	Year 2	3.16	.35	-.517	1.57
	Year 3	3.19	.37	-.587	.93
	Year 4	3.18	.35	-.758	1.82
Subjective economic satisfaction^*^		2.98	.953	.251	-.790
Residential environment satisfaction^*^		2.44	.814	.895	.464
Family relationship satisfaction^*^		2.01	.665	1.050	3.197
Social relationship satisfaction^*^		2.17	.624	.877	1.934
Subjective health status^*^		2.45	.866	.823	.451
Leisure life satisfaction^*^		2.63	.870	.521	-.231
Perceived income inequality		3.84	.856	-.723	.468

*Inverse coding

### 3.2. Analysis of the development trajectory of self-esteem

To determine an appropriate function to describe the temporal changes in self-esteem, this study conducted an analysis to evaluate the fit of three models: no change, linear, and non-linear. Ultimately, the linear growth model was deemed the most suitable based on the following fit indices: *x*^²^ =  15.848 (*p* < .000), CFI = .967, TLI = .959, RMSEA = .059, Akaike information criterion =  58.781, and Bayesian information criterion =  58.824. Consequently, a linear change model was employed to explore the initial levels and rates of change in the developmental trajectory of adults’ self-esteem.

In the linear change model, the mean initial level of self-esteem was 3.153, with a variance of.063, both of which were found to be statistically significant (*p* < .001). This suggests that the mean self-esteem score at the first time point was 3.153, and there were significant differences in self-esteem levels among individuals at this time. The average rate of change in self-esteem was.041, indicating that self-esteem increased by.041 points at each time point (*p* < .001). The variance in the rate of change was.034, signifying individual variability in the change in self-esteem over time. Moreover, the covariance between the initial level of self-esteem and the rate of change was -.017 (*p* < .001), demonstrating a negative relationship. This implies that individuals with initially high self-esteem exhibited a slower increase in self-esteem over time, while those with initially low self-esteem experienced a more significant increase (see Table 3).

**Table 3:** Development trajectory of self-esteem: Linear model analysis results.

	Mean (S.E.^a^)	Variate (S.E.)
Initial level (intercept)	3.153(.009)^***^	.063(.004)^***^
Rate of change (slope)	.041(.011)^***^	.034(.007)^***^
Initial level – Rate of change Covariance	-.017^***^	

^a^S.E. =  Standardized Estimates.

*p < .05,

**p < .01, and

***p < .001.

### 3.3. Research model verification results

This study analyzed the research model to investigate how subjective exclusion impacts changes in self-esteem through perceived income inequality. First, the fit of the model was assessed, resulting in χ^2^/df =  3.422, *p* < .001, RMSEA = .041, CFI = .963, and TLI = .949, indicating excellent fit overall.

Results of the research model analysis are presented in Table 4. Subjective exclusion had a positive effect on the mediator variable, perceived income inequality (*β* = .088, *p* < .01), while negatively affecting the dependent variable, initial self-esteem (*β* =  − .483, *p* < .001). Additionally, subjective exclusion had a significantly positive impact on the rate of change in self-esteem (*β* = .314, *p* < .01), indicating that higher levels of perceived subjective exclusion were associated with lower initial self-esteem levels that diverged over time.

**Table 4:** Analysis results of the research model.

Path	Standardized coefficient (S.E.^a^)	Non-standardized coefficient
Subjective exclusion → Perceived income inequality	.088(.044)	.128^**^
Subjective exclusion → Initial level of self-esteem	-.483(.018)	-.185^***^
Subjective exclusion → Rate of change in self-esteem	.314(.020)	.064^**^
Perceived income inequality → Initial level of self-esteem	-.064(.009)	-.017
Perceived income inequality → Rate of change in self-esteem	.232(.012)	.033^**^
Poverty status → Initial level of self-esteem	-.221(.020)	-.158^***^
Education level → Initial level of self-esteem	.264(.005)	.048^***^

^a^S.E. =  Standardized Estimates.

*p < .05,

**p < .01, and

***p < .001.

Perceived income inequality did not have a significant effect on the initial level of self-esteem but did have a positively significant impact on the rate of change in self-esteem (*β* = .232, *p* < .01).

When considering the control variables, low-income status was found to negatively impact the initial level of self-esteem (*β* =  − .221, *p* < .001), while education level had a significantly positive effect on initial self-esteem levels (*β* = .264, *p* < .001). However, as these control variables did not have a significant impact on the rate of change in self-esteem, the corresponding path was removed from the model (Table 4).

### 3.4. Verification of the indirect effects of the research model

The bootstrapping method was used to analyze the indirect effects of the research model, and the following results were obtained (refer to Table 5). The impact of subjective exclusion on the initial level of self-esteem through perceived income inequality was found to be negative (β =  -.006, p < .05). Subjective exclusion was positively associated with the rate of change in self-esteem through perceived income inequality (*β* = .021, *p* < .05) with statistical significance. In essence, subjective exclusion further strengthened the negative influence on both the initial level of self-esteem and rate of change. Thus, the mediated (indirect) pathway involving subjective exclusion, perceived income inequality, and self-esteem exhibited a partial mediating effect. Also, the proportion of indirect effects relative to total effects was calculated. The results indicated that the indirect effect of subjective exclusion on the initial level of self-esteem, mediated by perceived income inequality, accounted for 1.23% of the total effect. Likewise, the indirect effect on the rate of change in self-esteem was 6.29% of the total effect, highlighting the notable mediating role of perceived income inequality in the dynamic change of self-esteem.

**Table 5:** Effect decomposition of the research model.

Variables		Direct effects	Indirect effects	Total effects	SMC ^a^	Indirect effect (%) ^b^
Subjective exclusion → Perceived income inequality		.088 (.011)		.088 (.011)	.008 (.007)	
Initial level	Subjective exclusion → Self-esteem	-.483 (.007)	-.006 (.049)	-.489 (.007)	.386 (.016)	1.23
	Perceived income inequality → Self-esteem	-.064 (.098)		-.064 (.098)		
	Poverty status → Self-esteem	-.221 (.004)		-.221 (.004)		
	Education level → Self-esteem	.264 (.012)		.264 (.012)		
Rate of change	Subjective exclusion → Self-esteem	.314 (.019)	.021 (.020)	.334 (.016)	.165 (.023)	6.29
	Perceived income inequality → Self-esteem	.232 (.034)		.232 (.034)		

^a^SMC =  Squared multiple correlations.

^b^Proportion of Indirect Effect =  (Indirect Effect/ Total Effect) ×  100.

## 4. Discussion

This study examined how an individual’s perceived subjective exclusion influences their self-esteem development trajectory through perceived income inequality. First, higher perceived subjective exclusion was associated with higher levels of perceived income inequality, which acted as a mediator. Additionally, higher perceived subjective exclusion was linked to lower initial levels of self-esteem, with the gap widening over time. This supports Tadjoeddin’s research [16], which verified the relationship between exclusion and income inequality, and the results of previous research that examined the negative relationship between exclusion and self-esteem [43–45]. In Arslan’s study [43], social exclusion was found to be a factor that can predict low self-esteem, and Bernstein and Claypool [44] noted that the experience of social exclusion increases the distance between individuals and can cause emotional pain, including self-esteem issues. This study contributes to the existing literature by highlighting the mediating role of perceived income inequality in the relationship between subjective exclusion and self-esteem trajectories over time.

Second, while perceived income inequality did not significantly impact the initial level of self-esteem, it did have a positive effect on the rate of change in self-esteem, indicating a broad range of changes. This result is similar to that of previous studies that verified the influence of income inequality on self-esteem [28–31]. Control variables, such as low-income status and education level, significantly influenced the initial level of self-esteem but did not affect the rate of change.

These findings indicate that subjective exclusion, through perceived income inequality, plays a crucial role in shaping the trajectory of self-esteem over time. Accordingly, the implications are as follows. First, interventions that address adults’ self-esteem while considering social exclusion are necessary. In this study, both the initial value and rate of change in self-esteem presented significant changes. This means that temporal changes in self-esteem are valid even in adults and can be influenced by perceived social exclusion and income inequality. Previous research has shown that self-esteem tends to increase consistently until late adulthood [45]. Therefore, strategies must be implemented to enhance self-esteem across different life stages, with particular attention to vulnerable groups at risk of social exclusion. Such interventions should include educational programs that promote awareness of income inequality and social exclusion and offer practical tools to strengthen self-esteem and coping mechanisms.

For individuals in their 20s and 30s, who may experience low self-esteem due to economic pressures and the demands of various life tasks [46], programs aimed at developing self-esteem should be integrated into university curricula and workplace training. These programs can help young adults navigate career and relationship challenges more effectively. For those in their 40s and 50s, initiatives at the workplace and community level can address the psychological burdens associated with work-family conflict and caregiving responsibilities. As self-esteem has been identified as a predictor of success and well-being in various life domains, including relationships, work, and health [47–49], it is critical to design tailored interventions that reduce perceptions of social exclusion and income inequality while fostering a positive self-concept throughout adulthood.

Second, it is necessary to prepare more robust social and institutional mechanisms to reduce the likelihood of individuals experiencing exclusion. In this study, subjective exclusion negatively affected adults’ self-esteem. This implies that the exclusion experienced in life is not just a simple experience but may function as a risk factor that can lower an individual’s self-esteem. Prices have recently continued to increase globally, and class polarization has been intensifying. Although the need for medical care and safety has increased over the past three years due to COVID-19, welfare blind spots remain. When policymakers address social exclusion, they primarily base their approach on objective indicators, meaning that social systems cannot meet the subjective needs of each area. Park et al. [3] found that some individuals experienced exclusion in certain areas even though their socioeconomic activities and income status were stable. This finding implies that the concept of social exclusion should not simply be viewed as a concept of economic deprivation but should be considered from a multidimensional perspective. Since it is challenging to address these socio-environmental impacts at a micro level, a government-level approach is necessary. As mentioned earlier, as there has been little consideration for subjective exclusion within social exclusion, establishing objective and subjective factors within exclusion through continuous research and data construction is essential. Based on this point, there should be discussions on measures to improve systems that can alleviate the risk of exclusion in each area of life.

Third, it is necessary to develop national intervention strategies and implement effective legislation that can reduce income inequality in the Korean society. This study established that the experience of subjective exclusion had a significant effect on the rate of change in self-esteem through perceived income inequality. This implies that the higher the perceived subjective exclusion, the greater the gap in income perception and the lower one’s self-esteem. According to Lee and Kim [50], when the issue of income inequality is recognized in the Korean society, it is expected that the government will promote redistribution policies with greater responsibility. This indicates that the role of the government is more critical than that of individuals regarding income. Instability within the job market has affected individuals in their 20s and 30s, such as via the employment of contract workers and resultant wage gaps [51]. Those in their 40s and 50s are also highly likely to experience difficulties due to retirement, a poor social safety net, and the double burden of caring for their parents and raising their children. Thus, it is necessary to prepare and expand income class-based support policies that can alleviate income inequality.

Finally, this study has some limitations. It was unable to assess the actual gap in objective exclusion, as it focused solely on subjective exclusion and developed a research model. If future studies were to investigate both objective and subjective exclusion in their analysis, a more robust discussion could emerge regarding the connection between multidimensional exclusion and self-esteem, a key predictor of mental health. Additionally, although an individual’s current status as a student or employee was not directly included as a control variable, the highest level of education was controlled, serving as a proxy for this factor. However, adults who are still in school may experience different patterns of exclusion and self-esteem development compared to those who are employed or not engaged in work or study. Future research should consider these status-related factors to provide a more comprehensive understanding of the dynamics influencing self-esteem.

## 5. Conclusions

This study examined how perceived subjective exclusion in the Korean society affects the development of self-esteem through perceived income inequality. The research found that an individual’s subjective perceptions of socio-economic factors, including exclusion and income inequality, have a significant influence on self-esteem over time. Based on these results, the study proposed interventions to address inequalities in the social structure and promote greater awareness of subjective experiences, ultimately improving mental well-being for individuals.

## Supporting information

S1 Data Minimal_Dataset.
(XLSX)

S1 File (DOCX)
