# Supplementary material for: A longitudinal study on the impact of subjective exclusion on changes in self-esteem: the mediating effect of perceived income inequality
Source: PLoS One. 2025 Apr 2;20(4):e0321271. doi: 10.1371/journal.pone.0321271 (PMC11964206; doi:10.1371/journal.pone.0321271)
Supplement: S1 File — (DOCX) [file pone.0321271.s002.docx]

**Supporting information**

The data used in this study are from the Korean Welfare Panel Study (https://www.koweps.re.kr). This dataset is owned and managed by the Korea Institute for Health and Social Affairs (KIHASA) and requires a formal application for access due to legal and ethical restrictions. Researchers can request access via the KOWEPS website. The minimal dataset required to replicate the findings, including descriptive statistics, is provided in Table 2 within the manuscript. Due to data privacy concerns, we cannot provide raw data as Supporting Information. Additionally, S1_Minimal_Dataset.xlsx has been provided as Supporting Information, containing descriptive statistics of key variables used in the study.
